# The Effect of Erythropoietin on Ischemia/Reperfusion Injury after Testicular Torsion/Detorsion: A Randomized Experimental Study

**DOI:** 10.1155/2013/351309

**Published:** 2013-03-31

**Authors:** Fahimeh Kazemi Rashed, Babollah Ghasemi, Hamid Deldade Mogaddam, Mehran Mesgari

**Affiliations:** ^1^Department of Urology, Faculty of Medicine, Tabriz University of Medical Sciences, Tabriz 5166614756, Iran; ^2^Department of Pathology, Faculty of Medicine, Tabriz University of Medical Sciences, Tabriz 5166614756, Iran; ^3^Drug Applied Research Center, Tabriz University of Medical Sciences, Tabriz 5165665811, Iran

## Abstract

This study was conducted to investigate the protective effect of erythropoietin (EPO) on ischemia/reperfusion related changes after testicular torsion/detorsion. In a randomized experimental trial 30 male rats were randomly allocated into six equal groups of five rats each. Group I (orchiectomy for histopathologic examination), group II (sham operation), group III (torsion for 2 hours, and ischemia/detorsion for 24 hours, and orchiectomy); group IV (torsion for 2 hours, ischemia/detorsion for 24 hours with erythropoietin injection then orchiectomy), group V (torsion for 2 hours and detorsion and EPO injection and orchiectomy 1 week later, group VI (torsion for 2 hours/detorsion and orchiectomy 1 week later). Two groups (groups 4 and 5) received different protocols of erythropoietin administration after testicular torsion/distortion. other groups were not receiving erythropoietin. Johnsen's spermatogenesis scoring method and Cosentino's histologic staging method were used to assess main outcome measures of the study. After the experimentation, Johnsen's score in EPO Groups was statistically different from the score in some groups not receiving erythropoietin. Cosentino's score in EPO groups was statistically different from the score in all groups not receiving erythropoietin. Neovascularization, vascular necrosis, vascular congestion, edema, hemorrhage, and acute inflammation were observed in some groups. This study shows short-term protective efficacy of erythropoietin on rat testicular injury after ischemia/reperfusion.

## 1. Introduction 

Testicular torsion is the twisting of the spermatic cord, which cuts off the blood supply to the testicle and surrounding structures. It is more common during infancy and early adolescence. It is a very painful condition, but mainly it is a surgical emergency because it may result in the loss of the affected testicle if not treated promptly. Torsion is the most common cause of testicle loss in adolescent males. Some men may be predisposed to testicular torsion as a result of inadequate connective tissue within the scrotum. However, the condition can result from trauma to the scrotum, particularly if significant swelling occurs. It may also occur after strenuous exercise or may not have an obvious cause [1]. 

Surgery is usually required and should be performed as soon as possible after symptoms begin. If surgery is performed within 6 hours, most testicles can be saved. Testicular torsion and also the detorsion procedure induce morphological as well as biochemical changes caused mostly by ischemia/reperfusion injury in the testicular tissue [2].

 Dysfunction induced by free radicals is the major component of ischemic process due to torsion in the testis, and many studies focused on protective effects of special medications and erythropoietin [3, 4]. Many Animal studies have discovered a protective role for erythropoietin against the aftermaths of ischemia/reperfusion in various organ tissues including kidney, heart, liver, and ovary tissues in animals and nervous system [5–11]. Few studies have recently been conducted on testicular tissues either [12–15]. However, available knowledge is limited considering the methodological variations. The aim of this study was to investigate the protective effect of erythropoietin on ischemia/reperfusion related changes after testicular torsion/detorsion.

## 2. Materials and Methods

A study was conducted in 2008 in Urology Department of Imam Reza University Hospital in Tabriz, Iran. Animals were 200–300 gr weighted male wistar rats that were breeded in Razi institute of Iran and kept, prior and through the investigation process, under normal and similar light, temperature, and feeding plan in the animal lab in Drug Applied Research Center, Tabriz University of medical sciences. In a randomized experimental trial 30 rats were randomly allocated into six equal groups of five rats each. Both testes of each rat were studied. The groups were treated as follows.

*Group 1*. Both testes of the five studied rats were orchidectomized and sent for histopathology examination. 

*Group 2*. All five rats underwent lower abdominoscrotal incision (because a wide inguinal canal the testes move freely between abdomen and scrotum) and orchiopexy. After 24 hours both testes of the studied rats were orchidectomized and sent for histopathology examination.

*Group 3*. All five rats underwent lower abdominoscrotal incision. They received 720 degrees bilateral torsion for two hours (based on other studies) then detorsion and orchiopexy were done on them [12, 16]. After 24 hours both testes of the studied rats were orchidectomized and sent for histopathology examination. 

*Group 4*. All five rats underwent lower abdominoscrotal incision. They received 720 degrees bilateral torsion for two hours then detorsion and orchiopexy were done on them. Then erythropoietin was injected and after 24 hours both testes of the studied rats were orchidectomized and sent for histopathology examination. 

*Group 5*. All five rats underwent lower abdominoscrotal incision. They received 720 degrees bilateral torsion for two hours and then detorsion and orchiopexy were done on them. Then erythropoietin was injected, and after one week both testes of the studied rats were orchidectomized and sent for histopathology examination. 

*Group 6*. All five rats underwent lower abdominoscrotal incision. They received 720 degrees bilateral torsion for two hours, and then detorsion and orchiopexy were done on them. After one week both testes of the studied rats were orchidectomized and sent for histopathology examination. 

Prior to incisions general anesthesia was induced using 2.5 mg/kg midazolam as intraperitoneal injection. Erythropoietin was administered intravascular in a dose of 3000 u/kg in groups 4 and 5. The pathologist and evaluator were blind to the type of intervention. Spermatogenesis was assessed using Johnsen's spermatogenesis scoring method which is based on the concept that testis damage causes a successive disappearance of the most mature germ cell type [17]. Following is the scoring grades:

 score 10: complete spermatogenesis with regular tubules;
 score 9: many sperms, irregular germinal epithelium;
 score 8: few sperms;
 score 7: no sperms, many spermatids;
 score 6: few spermatids;
 score 5: no sperm or spermatids;
 score 4: few spermatocytes;
 score 3: presence of spermatogonia;
 score 2: presence of Sertoli's cells;
 score 1: no cells.

Histological assessments were based on Cosentino's histologic staging method [18]. 

Data were gathered and analyzed using SPSS version 15 statistical software package. Measures of continuous scales were compared using Kruskall-Wallis test followed by Mann-Whitney *U* test as post hoc. Categorical variables were assessed using contingency tables and chi-squared or Fisher's exact tests. *P* < 0.05 was considered a statistical significance level in primary tests. Bonferroni correction was used for qualitative parameters in multiple comparisons. 

Study was approved by committee of ethics in Tabriz University of medical sciences. Code of ethics on working with lab animals was followed in this research and interdisciplinary principles and guidelines for the use of animals in research were considered. 

## 3. Results and Discussion 

**Figure 1:**
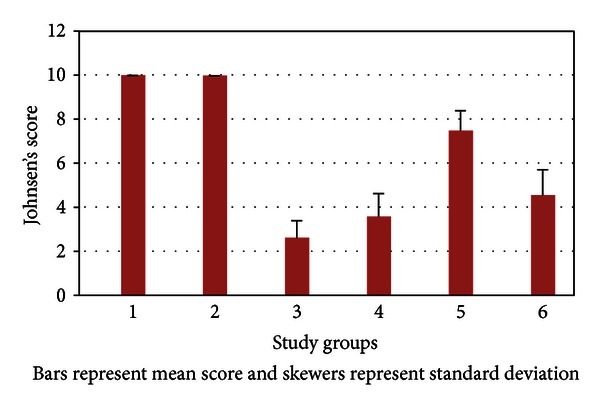
Mean Johnsen's score compared among the study groups.

Median Johnsen's score was the highest among rats in group 5 when compared to other rats except control rats in groups 1 and 2 that had not undergone testicular torsion. Mean (SD) Johnsen's score is compared among the groups in Figure 1. Medians of Johnsen's score were 10, 10, 2, 3.5, 7.5, and 5 in groups 1–6, respectively. 

In pairwise statistical assessment of comparisons, it was found that Johnsen's score in group 5 was statistically different from the score in all groups not receiving erythropoietin. However, Johnsen's score in group 4 was statistically different from the score in groups 1 and 2 not receiving erythropoietin. Further details are given in Table 1. 

**Figure 2:**
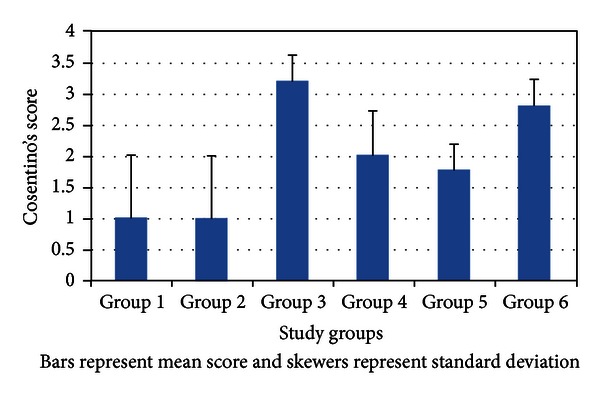
Mean Cosentino's score compared among the study groups.

Mean (SD) Cosentino's score is compared among the groups in Figure 2. Medians of Cosentino's score were 1, 1, 3, 2, 2, and 3 in groups 1–6, respectively.

In pairwise statistical assessment of comparisons, it was found that Cosentino's score in group 5 and 4 was statistically different from the score in all groups not receiving erythropoietin. Further details are given in Table 1.

**Figure 3:**
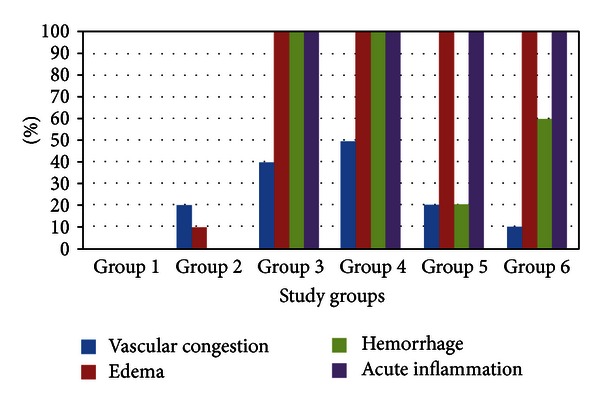
Relative frequency distribution of vascular congestion, edema, hemorrhage, and acute inflammation compared among study groups.

Statistical assessment of comparisons made between erythropoietin groups and other four control groups found that the frequency distribution in vascular congestion was statistically different only when group 4 was compared with group 1 (*P* = 0.03); the difference in frequency distribution of neovascularization and vascular necrosis was not statistically significant for any of the comparison pairs; groups 4 and 5 had significantly different frequency of edema only with groups 1 and 2 (*P* = 0.001); hemorrhage frequency was different when comparing group 4 with groups 1 and 2 (*P* = 0.001), and also the frequency difference of hemorrhage between groups 5 and 3 was also statistically significant (*P* = 0.001); groups 4 and 5 had significantly different frequency distribution of acute inflammation only with groups 1 and 2 (*P* = 0.001) (Figure 3).

The protective effects of erythropoietin on renal and cardiovascular ischemic injuries are shown earlier [5, 11, 19]. Effect of erythropoietin on gonadal ischemia is also shown to be positive relieving ischemic injuries after ovarian torsion in rat and mouse models [6, 8, 9, 20]. 

In this study we assessed the effect of erythropoietin on ischemic/reperfusion changes after testicular torsion. The results of present study were indicative of efficacy of erythropoietin in relieving the changes after ischemia/reperfusion when compared with similar control groups that had not received erythropoietin. The study also found that seven days after ischemia/reperfusion both Johnsen's score and histological grading were significantly better in groups receiving erythropoietin. 

The first study on effects of erythropoietin on testes injury was published in 2005 by Dobashi et al. They investigated the effects of rat erythropoietin on spermatogenesis by transferring rat Epo gene into cryptorchid testes by means of in vivo electroporation and found that it may reduce the risk of the germ cell loss caused by cryptorchidism [21]. Later in 2007 Yazihan et al. in their study in a five-group rat study found that erythropoietin has antiapoptotic and anti-inflammatory effects following testicular torsion [14]. Two other studies on rat model showed also the positive effect of erythropoietin on ischemic testis injuries [12, 22]. It was found that erythropoietin can decrease cell damage and apoptosis. Köseoğlu et al. concludes that erythropoietin preserves the intact somniferous tubular morphology, lowers the percentage of necrotic seminiferous tubules, and reduces the histological damage [13]. One study used mouse model to assess effect of erythropoietin, but as darbepoetin *α*, on ischemia caused by testis torsion finding that it affects histological grading. The mentioned study assessments were done four hours after detorsion [15].The major methodological variations in studies conducted on effect of erythropoietin after gonadal ischemia include gonadal type as ovaries versus testes, animal type as rat versus mouse, drug administration form as oral versus parenteral, assessment timing, and drug administration timing and order such that nearly every study had some exclusive methodological aspects. However, regardless of methodological variations, our findings on efficacy of erythropoietin on aftermaths of gonadal ischemia/reperfusion were consistent with available literature. 

## 4. Conclusions

Based on our findings and available knowledge, it can be inferred that erythropoietin has positive effects on gonadal ischemia/reperfusion injury. Specifically we conclude supporting short-term efficacy of erythropoietin on rat testicular injury after ischemia/reperfusion. However, available knowledge, including our findings, has not yet turned complete. Two major areas of necessary future research could be dose-response studies and studying time variance of erythropoietin efficacy in this regard. 

## Figures and Tables

**Table 1:** *P* values for pairwise statistical comparisons of median Johnsen's score and Cosentino's score in groups of rats receiving erythropoietin versus control groups.

Control groups	Erythropoietin groups			
	Group 4		Group 5	
	Johnsen's score	Cosentino's score	Johnsen's score	Cosentino's score
Group 1	*P* = 0.001	*P* = 0.002	*P* = 0.001	*P* = 0.001
Group 2	*P* = 0.001	*P* = 0.002	*P* = 0.001	*P* = 0.001
Group 3	*P = 0.035	*P* = 0.002	*P* = 0.001	*P* = 0.001
Group 6	*P = 0.063	*P* = 0.015	*P* = 0.001	*P* = 0.001

*Not statistically significant. Only *P* values lower than 0.003 were considered as statistically significant.
